# The Immediate Effect of Cold Spinal Spray and Cold Spinal Bath on Cognition Among Young Adults: A Three-Armed Randomized Controlled Trial

**DOI:** 10.7759/cureus.104034

**Published:** 2026-02-21

**Authors:** Avishee Sinha, Sujatha KJ

**Affiliations:** 1 Natural Therapeutics, Shri Dharmasthala Manjunatheshwara (SDM) College of Naturopathy and Yogic Sciences, Ujire, IND

**Keywords:** autonomic function, cognitive function, cold spinal bath, cold spinal spray, heart rate variability (hrv), hydrotherapy, mental cognition, naturopathy, randomized controlled trial (rct), vagus nerve stimulation

## Abstract

Background

Cognition is a complex process that involves acquiring knowledge and understanding. Hydrotherapy, a part of naturopathy, particularly cold spinal spray and cold spinal bath, is used to influence physiological responses. However, its immediate effects on cognition remain unexplored. This study aims to evaluate the immediate effect of cold spinal spray and cold spinal bath on cognition among young adults using a three-armed randomized controlled trial.

Methods

A total of 90 healthy volunteers (aged 18-25 years) were recruited and randomly assigned into three groups: group 1 - cold spinal spray (n=30), group 2 - cold spinal bath (n=30), and group 3 - control group (n=30). Interventions were administered for 15 minutes. Cognitive assessments were conducted pre- and post-intervention using the Stroop test and Letter-Digit Substitution Test (LDST). Heart rate variability (HRV) was measured as a secondary outcome. Data were analyzed using Jamovi version 2.7.6 statistical software (the Jamovi Project, Sydney, Australia).

Results

Both cold spinal spray and cold spinal bath groups demonstrated statistically significant improvements in cognitive performance, including increased accuracy and reduced error rates, compared with the control group (p<0.05), with large effect sizes observed for primary cognitive outcomes (η²≥0.14). The cold spinal spray group exhibited a more pronounced improvement in cognitive scores compared to the cold spinal bath group. HRV analysis showed enhanced autonomic regulation post-intervention most in the cold spinal spray group.

Conclusion

Cold spinal spray and cold spinal bath have an immediate positive effect on cognitive functions, with cold spinal spray demonstrating a slightly greater impact. These findings suggest the potential of hydrotherapy in enhancing cognition and the scope of these applications to patients with cognitive decline.

## Introduction

Cognition involves acquiring knowledge through thought, experience, and senses [1]. Sensory data is integrated across brain regions, including limbic areas for emotion and memory [2]. Key cognitive domains-memory, attention, executive function-are influenced by mood and motivation and assessed via neuropsychological tests [3]. Memory, especially working memory, involves retaining and manipulating information, assessed by formal tests. Healthy lifestyle habits slow memory decline by reducing vascular risk and inflammation [4]. Naturopathic medicine, akin to primary care, emphasizes lifestyle counseling, with hydrotherapy using water's physical properties for pain relief, temperature regulation, and circulation [5-6].

Emerging research indicates that cold exposure positively influences cognitive performance by increasing the release of neurotransmitters and reducing fatigue [7]. Specifically, cold-water immersion has been shown to decrease sleepiness and enhance cognitive functioning during sleep inertia-the transitional groggy state following awakening [8]. Adkins and colleagues emphasize the relevance of this intervention for individuals such as military personnel and first responders who require immediate cognitive readiness upon waking [9]. Furthermore, repeated cold exposure, known as cold acclimation, has been reported to improve adaptation to cold-induced cognitive challenges. Research by Jones et al. suggests that regular cold exposure fosters mental resilience and enhances cognitive function under such stressors [10].

In addition to cognitive enhancements, cold therapy triggers physiological responses that support brain health. It elevates norepinephrine production, which in turn increases dopamine levels-a neurotransmitter vital for motivation, learning, and task performance [11]. Cold exposure also induces production of the cold shock protein RBM3, crucial for synaptic maintenance and repair. Higher RBM3 levels are associated with neuronal regeneration and synaptic restoration, potentially offering protection against cognitive decline and neurodegenerative diseases [12].

Spinal spray tub is an equipment which is made up of fiberglass material with a perforated tube in the middle of the tub; this tub contains 40 liters of water that circulates and is connected with a 0.5HP motor that's adjusted below the tub. This tub can circulate cold, neutral, and hot water according to different temperatures as required for patients [13]. Spinal bath is a local, non-pressurized hydratic measure in which the spinal area is exposed to water of a certain temperature for a specific duration to get desired effects. This procedure influences all organs of the body, since most of the nerve roots start from the spinal cord. They are the sensory centers, temperature controlling centers, vasomotor centers, and sympathetic and parasympathetic centers in the brain and spinal cord. The small and large blood vessels of the heart, lungs, digestive system, and brain contract or dilate depending on the temperature of the water used in the spinal bath. Spinal bath also improves oxygen supply to the lungs and heart by increasing blood circulation [14].

While prior studies have evaluated the effect of cold spinal bath and cold spinal spray on autonomic functions in healthy individuals, their direct impact on cognition remains unexplored. This study aims to evaluate the immediate effects of cold spinal spray and cold spinal bath on cognitive performance in healthy young adults using a three-arm randomized controlled design compared to a control group with validated cognitive assessments, and to assess the occurrence of any intervention-related adverse effects. Results may deepen understanding of hydrotherapy's role in cognitive enhancement and promote natural interventions for mental well-being.

## Materials and methods

Methods

This three-armed randomized controlled study at one of the colleges in South India, conducted during the month of December 2024, included 90 participants, selected from 120 screened, based on G-Power analysis recommending 30 per group for 95.0% confidence and 80.0% power, α=0.05, ensuring reliable results across cold spinal spray, cold spinal bath, and control groups [15]. No interim analyses or stopping guidelines were planned or performed.

Participants

A total of 90 healthy young adults (aged 18-30 years) were randomly assigned into three groups: cold spinal spray (n=30), cold spinal bath (n=30), and control (n=30). Participants aged between 18-30 years were included from both genders [15], who were able and willing to provide consent. Individuals with diabetes, cancer, hypertension, anxiety, or depression [16], history of cold intolerance or allergy to cold, usage of psychotropic medications or substances affecting cognition or autonomic function [17], complaint of infections or open wounds, enrolled in any other research trial within the previous three months, suffered acute illness within two weeks prior to study entry and female subjects during menstruation, those who are under any medications during the study were excluded to ensure healthy sample [18]. Informed consent was obtained and secured from all participants. Institutional ethical clearance was obtained, and the trial was further registered with the Clinical Trial Registry of India (CTRI/2024/07/070437 10th July 2024), and informed consent was secured, with no protocol amendments after trial commencement. Participants of both sexes were randomized via a computer-generated simple random sequence by an independent investigator via an open list of random numbers. The enrolling person had no access to the sequence, and outcome assessors and data analysts were blinded to group allocation, while participant and provider blinding were not feasible due to the nature of the interventions. The Consolidated Standards of Reporting Trials (CONSORT) 2017 guidelines were followed [19].

Study design

**Figure 1:**
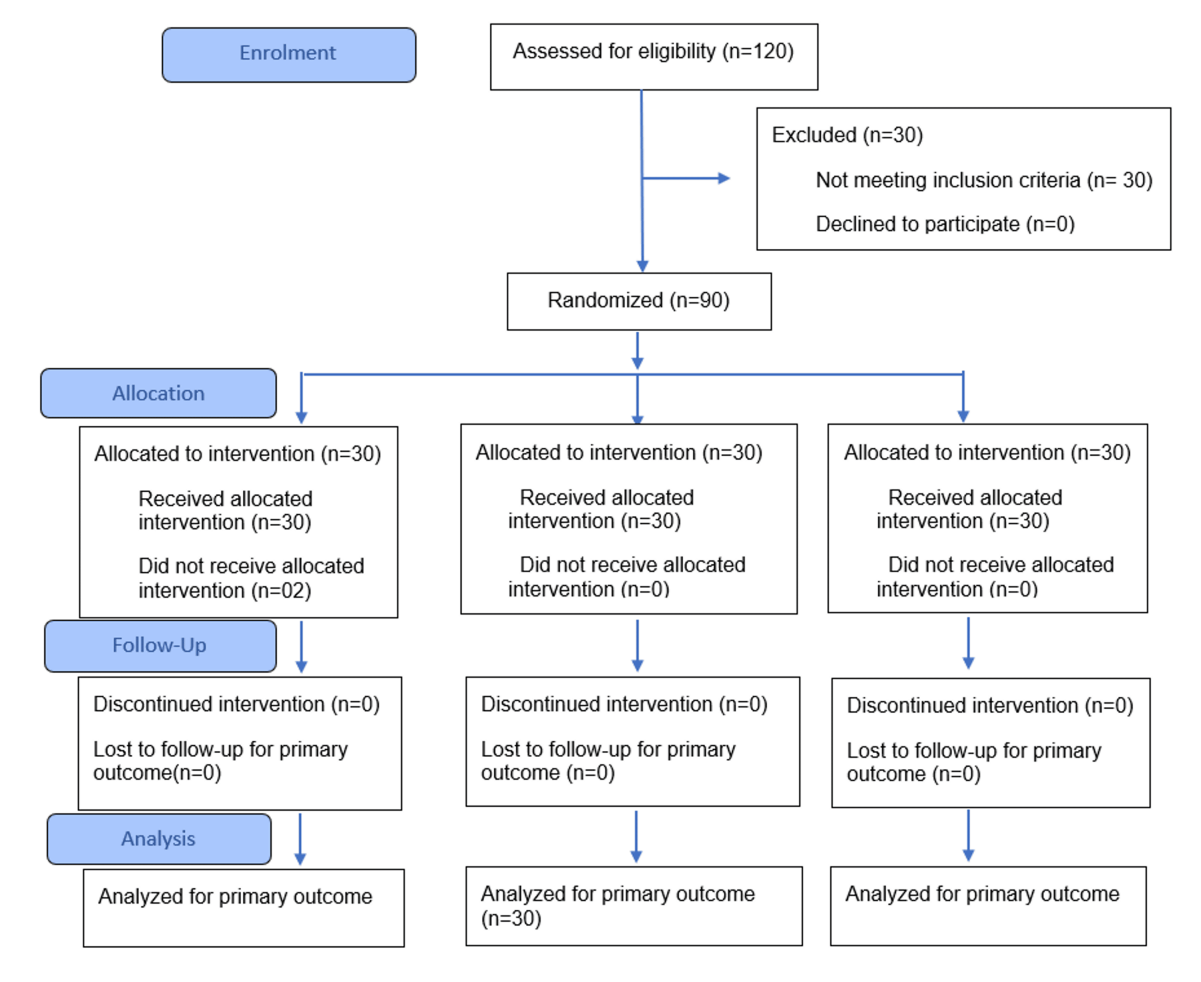
CONSORT flow chart of the study CONSORT - Consolidated Standards of Reporting Trials

This was a single-center single-blinded parallel-group three-arm randomized controlled trial with 1:1:1 allocation, designed under a superiority framework. The trial ended as planned after completion of participant recruitment and immediate post-intervention outcome assessment, with no early stopping or termination. The participant recruitment, allocation, follow-up, and analysis are shown in Figure 1.

Intervention

Group 1, group 2, and the control group received a cold spinal spray, a cold spinal bath, and a supine rest, respectively. The cold spinal spray group received a 15-minute intervention using a perforated tube that sprayed cold water (18-24°C) directly onto the spine while lying supine [20-21]. The cold spinal bath group underwent a 15-minute immersion, where they lay in a shallow tub with cold water (18-24°C) covering the spine from the nape of the neck to the lower back [14,20]. All participants completed the assigned intervention as allocated, adherence was complete, and no protocol deviations occurred.

Bachelor of Naturopathy and Yogic Science (BNYS) doctors supervised both interventions for safety and consistency, and potential harms related to cold exposure were predefined as any adverse physical or autonomic symptoms, including dizziness, discomfort, cold intolerance, skin reactions, or syncope, and assessed systematically through direct monitoring during the intervention and participant enquiry immediately post-intervention. The control group rested supine for 15 minutes without treatment. Participants avoided caffeine and strenuous activity for 24 hours prior. Water temperature was monitored using a digital thermometer, and interventions took place in a controlled setting to minimize external cognitive influences. The treatment was given in the morning from 7 to 9 am after about 12 hours of fasting, with minimal clothing, and no concomitant care was provided during the trial.

Outcome measures

The primary outcome of this study was cognitive performance, assessed using two validated tests: the Stroop test and the Letter-Digit Substitution Test (LDST). The Stroop test measured cognitive inhibition and processing speed by evaluating reaction time and accuracy in identifying ink colors of mismatched words using Inquisit software version 7 [22-23]. The LDST assessed attention, processing speed, and working memory by requiring participants to substitute digits for corresponding letters within 60 seconds [24]. For both cognitive tests, outcomes were analyzed as change from baseline (post-intervention minus pre-intervention values), and assessed at two time points: pre-intervention and immediately post-intervention. The secondary outcome was autonomic function, assessed through heart rate variability (HRV) using an electrocardiogram (ECG). ECG was recorded using 8 - a channel human physiology system (Power Lab 8/35, AD Instruments, Australia) - and acquired using the limb Lead II system, i.e., the electrodes were placed on the right arm and both legs. HRV analysis included time-domain and frequency-domain parameters such as standard deviation of R-R interval (SDNN), square root of the mean of the sum of the squares of differences between adjacent RR intervals (RMSSD), low frequency (LF), high frequency (HF), and LF/HF ratio to evaluate sympathetic and parasympathetic nervous system balance, analyzed as change from baseline at pre-intervention and immediately post-intervention time points. The HRV spectrum is believed to be a useful indicator of cardiac sympathetic activity (reflected by LF band power values) and parasympathetic activity (reflected by HF band power values) [25].

Statistical methods

Data were analyzed in Jamovi software 2.7.6 (the Jamovi Project, Sydney, Australia). Normality was assessed (Shapiro-Wilk). Appropriate parametric or non-parametric tests compared groups: paired t-test, Wilcoxon, One Way ANOVA, Kruskal-Wallis. All statistical tests were two-tailed, and statistical significance was set at p-value <0.05. Data was arranged in mean ± SD, and results were tabulated and graphed. Post hoc analysis was performed using pairwise comparisons with Tukey's test to identify specific group differences in HRV parameters. All randomized participants who completed the intervention and outcome assessments were included in the analysis and retained in their originally allocated intervention groups. There were no missing data; therefore, no imputation methods were applied. No prespecified or post-hoc subgroup or sensitivity analyses were performed.

## Results

The study comprised 90 participants with a mean age of 19.10 ± 1.79 years. The mean ages of male and female participants were 18.94 ± 1.74 and 19.19 ± 1.82 years, respectively. The sample included 32 males (35.6%) and 58 females (64.4%) (Table 1).

**Table 1:** Baseline Characteristics of the subjects in the cold spinal bath (group 1), cold spinal Spray (group 2) and control (group 3) Data expressed in mean±SD Group 1 - cold spinal bath; group 2 - cold spinal spray; group 3 - control group

Variables	Group 1	Group 2	Group 3
Age (years)	17.73 ± 1.36	17.82 ± 1.39	18.04 ± 1.41
Gender	Male = 10; Female = 20	Male = 8; Female = 22	Male = 14; Female = 16

For all primary and secondary outcomes, all randomized participants (n=30) received the intended intervention and were included in the analysis, with no losses or exclusions; recruitment occurred in December 2024 with immediate pre- and post-intervention assessment, the trial ended as planned, interventions were delivered under BNYS doctor supervision with full adherence and no deviations, and no concomitant care was provided, complete outcome data were available for all participants at the post-intervention time point. Results showed that group 1 improved correct responses from 35.43 ± 6.14 to 41.33 ± 5.57, group 2 from 37.70 ± 6.11 to 43.06 ± 6.62, and group 3 from 35.13 ± 3.75 to 37.60 ± 4.74 (p=0.001***). The Stroop effect decreased in groups 1 and 2 but increased in group 3 (p=0.010**). Accuracy improved significantly in groups 1 (93.41 ± 5.71 to 97.14 ± 3.18) and 2 (91.15 ± 5.40 to 94.84 ± 8.37), with no significant change in errors (p=0.456) and error rate (p=0.156) across groups. Overall, significant improvements were seen in correct responses, Stroop effect, and accuracy, while errors and error rate did not differ significantly. The ANOVA results showed significant effects for correct responses (F=7.809, df1=2, df2=57, p=0.001***, η²=0.21), Stroop effect (F=5.028, df1=2, df2=57, p=0.010**, η²=0.15), and accuracy (F=5.256, df1=2, df2=50, p=0.009**, η²=0.16). No significant effects were found for errors (F=0.797, df1=2, df2=53, p=0.456) or error rate (F=1.927, df1=2, df2=51, p=0.156). This indicates significant group and time effects on cognitive performance measures related to correct responses, Stroop interference, and accuracy (Table 2).

**Table 2:** Baseline (T0) and post-test (T1) assessment with statistical analysis within (Wilcoxon signed-rank test) and between (one-way ANOVA-Kruskal-Wallis) the three groups for Digit Letter Substitution Test and Stroop task performance #<0.05, ##<0.01, ###<0.001 within the group; *<0.05, **<0.01, ***<0.001 between the group. Data expressed as mean±SD. Group 1 - cold spinal bath; group 2 - cold spinal spray; group 3 - control group

Variable	Group 1 pre (mean ± SD)	Group 1 post (mean ± SD)	Wilcoxon signed-rank test	Group 2 pre (mean ± SD)	Group 2 post (mean ± SD)	Wilcoxon signed-rank test	Group 3 pre (mean ± SD)	Group 3 post (mean ± SD)	Wilcoxon signed-rank test	p-value	F value	η²
Correct responses	35.43 ± 6.14	41.33±5.57	<0.001^###^	37.70 ± 6.11	43.06 ± 6.62	0.003^##^	35.13 ± 3.75	37.60 ± 4.74	0.03^#^	0.001***	7.809	0.215
Errors	0.16 ± 0.59	0.13 ± 0.34	0.75	0.13 ± 0.43	0.40 ± 1.19	0.25	0.10 ± 0.30	0.20 ± 0.40	0.29	0.456	0.797	0.027
Stroop effect	295.82 ± 440.44	237.58 ± 258.07	0.53	333.58 ± 536.13	315.74 ± 308.65	0.87	412.90 ± 278.92	429.13 ± 212.35	0.87	0.010**	5.028	0.150
Accuracy	93.41 ± 5.71	97.14 ± 3.18	0.06	91.15 ± 5.40	94.84 ± 8.37	0.20	90.67 ± 9.21	93.01 ± 6.49	0.26	0.009**	5.256	0.156
Error rate	9.41 ± 5.18	2.857 ± 3.182	1.00	8.15 ± 4.40	5.15 ± 8.37	1.00	9.32 ± 9.21	4.74 ± 5.55	0.02^#^	0.156	1.927	0.063

Pairwise comparisons revealed significant mean differences in correct responses between group 1 and group 3 (3.73, p=0.034**) and between group 2 and group 3 (5.47, p=0.001***), while group 1 vs group 2 was not significant (-1.73, p=0.470). For the Stroop effect, a significant difference was found only between group 1 and group 3 (-192, p=0.016**). Accuracy differences were significant between group 1 and group 3 (4.13, p=0.037**), but not in other comparisons. No significant differences were observed for errors or error rate across any group comparisons (all p>0.3) (Table 3).

**Table 3:** Post hoc analysis results for the Stroop task performance and Digit Letter Substitution Test p-value<0.001, *** : highly significant; ** : significant Group 1 - cold spinal bath; group 2 - cold spinal spray; group 3 - control group; F - degree of freedom; St - Sternberg test

Variable	Comparison	Mean difference	p-value
Correct	Group 1 vs group 2	-1.73	0.47
	Group 1 vs group 3	3.73	0.034**
	Group 2 vs group 3	5.47	0.001***
Errors	Group 1 vs group 2	-0.267	0.361
	Group 1 vs group 3	-0.067	0.937
	Group 2 vs group 3	0.2	0.562
Stroop effect	Group 1 vs group 2	-78.2	0.485
	Group 1 vs group 3	-192	0.016**
	Group 2 vs group 3	-113	0.222
Accuracy	Group 1 vs group 2	2.3	0.348
	Group 1 vs Group 3	4.13	0.037**
	Group 2 vs group 3	1.83	0.511
Error rate	Group 1 vs group 2	-2.3	0.313
	Group 1 vs group 3	-1.89	0.456
	Group 2 vs group 3	0.415	0.962

Post-intervention, RMSSD increased significantly (p=0.020) with group 1 rising from 49.35 ± 32.68 to 75.58 ± 41.31 ms and group 2 from 30.20 ± 12.37 to 47.83 ± 13.64 ms. HF (nu) also showed significant increases (p=0.030), with group 1 increasing from 1318.64 ± 1455.77 to 2047.84 ± 2477.49 and group 2 from 387.81 ± 198.11 to 886.76 ± 531.32. HF Power (%) improved significantly (p=0.002) in group 1 (37.98 ± 15.52 to 50.14 ± 14.40) and decreased in group 3. LF/HF ratio decreased significantly (p=0.001), with Group 1 lowering from 1.28 ± 1.32 to 0.60 ± 0.58 and group 2 from 0.92 ± 0.57 to 0.31 ± 0.18, indicating enhanced parasympathetic modulation. Other measures, including mean RR, SDNN, PNN50, and LF Power, showed no significant post-intervention differences (p > 0.05). The ANOVA results showed significant effects for RMSSD (F=3.890, df1=3, df2=27, p=0.020**), HF (F=3.384, df1=3, df2=32, p=0.030**), HF power (F=7.180, df1=3, df2=18, p=0.002**), and LF/HF ratio (F=9.596, df1=3, df2=34, p=0.001***). No significant effects were observed for mean RR (F=1.765, p=0.168), SDNN (F=2.211, p=0.112), PNN50 (F=1.119, p=0.364), or LF power (F=0.472, p=0.705). These indicate significant group differences in HRV parameters related to parasympathetic activity and sympathovagal balance post-intervention. Large effect sizes were observed for HF power (%) (η²=0.20) and LF/HF ratio (η² = 0.25), indicating substantial intervention-related effects on parasympathetic activity and sympathovagal balance. Medium-to-large effect sizes were noted for RMSSD (η² = 0.12) and HF (nu) (η² = 0.11), suggesting meaningful improvements in vagal modulation following the interventions. Medium effect sizes were observed for SDNN (η²=0.07) and LF (nu) (η²=0.09), while small effects were seen for MEAN RR (η²=0.06), PNN50 (η²=0.04), and LF power (%) (η²=0.02) (Table 4).

**Table 4:** Baseline (T0) and post-test (T1) assessment with statistical analysis within (Wilcoxon signed-rank test) and between (one-way ANOVA Kruskal-Wallis) the three groupsfor HRV #<0.05, ##<0.01, ###<0.001 within the group;*<0.05, **<0.01, ***<0.001 between the group. Data expressed as mean±SD. Group 1 - cold spinal bath; group 2 - cold spinal spray; group 3 - control group; HRV - heart rate variability; mean RR - average of R-R interval; SDNN - standard deviation of R-R interval; RMSSD - square root of the mean of the sum of the squares of differences between adjacent RR intervals; PNN50 - derived by dividing NN50 by the total number of RR intervals; LF - low-frequency band of HRV; HF - high-frequency band of HRV; LF/HF - ratio of low frequency to high frequency; SD - standard deviation

Variable	Group 1 pre (mean ± SD)	Group 1 post (mean ± SD)	Wilcoxon signed-rank test	Group 2 pre (mean ± SD)	Group 2 post (mean ± SD)	Wilcoxon signed-rank test	Group 3 pre (mean ± SD)	Group 3 post (mean ± SD)	Wilcoxon signed-rank test	Post p-Value	F value
MEAN RR (ms)	778.14 ± 130.99	689.19 ± 323.95	0.29	736.60 ± 36.68	802.60 ± 44.08	0.95	792.33 ± 109.97	730.64 ± 273.82	0.23	0.168	1.765
SDNN (ms)	50.24 ± 26.00	58.97 ± 30.16	0.17	32.73 ± 13.02	45.93 ± 10.85	0.24	64.06 ± 28.39	62.84 ± 23.88	0.73	0.112	2.211
RMSSD (ms)	49.35 ± 32.68	75.58 ± 41.31	0.01^#^	30.20 ± 12.37	47.83 ± 13.64	0.13	50.42 ± 34.34	43.61 ± 33.46	0.45	0.020**	3.890
PNN50 (%)	25.61 ± 26.09	35.63 ± 25.94	0.12	11.73 ± 12.50	22.25 ± 14.41	0.12	34.29 ± 24.59	35.99 ± 22.29	0.92	0.364	1.119
LF (nu)	950.92 ± 1015.76	1435.92 ± 1465.99	0.14	431.04 ± 337.51	759.84 ± 409.92	0.42	1174.14 ± 813.28	1462.93 ± 1162.33	0.30	0.063	2.777
HF (nu)	1318.64 ± 1455.77	2047.84 ± 2477.49	0.16	387.81 ± 198.11	886.76 ± 531.32	0.30	1787.33 ± 1565.82	1916.69 ± 1546.66	0.85	0.030**	3.384
LF Power (%)	31.12 ± 10.55	30.06 ± 9.72	0.61	33.05 ± 10.34	30.65 ± 7.75	0.67	33.19 ± 15.39	30.96 ± 12.83	0.63	0.705	0.472
HF Power (%)	37.98 ± 15.52	50.14 ± 14.40	0.007^##^	35.08 ± 16.56	41.63 ± 14.82	0.03^#^	38.65 ± 19.20	29.75 ± 17.86	0.07	0.002**	7.180
LF/HF Ratio	1.28 ± 1.32	0.60 ± 0.58	0.01^#^	0.92 ± 0.57	0.31 ± 0.18	0.04^#^	0.96 ± 0.66	1.44 ± 1.12	0.002^##^	0.001***	9.596

Pairwise comparisons showed a significant mean difference in RMSSD between group 1 and group 3 (31.98, p=0.011**), and in HF Power between group 1 and group 3 (20.4, p=0.001***). The LF/HF ratio difference was also significant between group 1 and goup 3 (-0.843, p=0.002**). No significant mean differences were observed for mean RR, SDNN, PNN50, LF nu, HF nu, or LF Power across any group comparisons (all p>0.1). These findings highlight significant improvements in parasympathetic modulation and sympathovagal balance primarily between groups 1 and 3 (Table 5). No harms or unintended events were observed or reported in any of the study groups during or after the intervention. 

**Table 5:** Post hoc analysis for HRV for each group p-value<0.001, *** : highly significant; * : significant; † : minimally significant M - mean; SD - standard deviation;

Variable	Group comparison	Mean difference	p-value
Mean RR	Group 1 vs group 3	-113.45	0.247
	Group 1 vs group 2	-123.21	0.177
	Group 2 vs group 3	9.75	0.999
SDNN	Group 1 vs group 3	-4.31	0.748
	Group 1 vs group 2	1.12	0.978
	Group 2 vs group 3	-5.43	0.698
RMSSD	Group 1 vs group 3	31.98	0.011**
	Group 1 vs group 2	27.7	0.432
	Group 2 vs group 3	12.6	0.598
PNN50	Group 1 vs group 3	-0.83	0.998
	Group 1 vs group 2	1.23	0.995
	Group 2 vs group 3	-2.06	0.961
LF nu	Group 1 vs group 3	3.23	0.563
	Group 1 vs group 2	0.76	0.919
	Group 2 vs group 3	1.98	0.768
HF nu	Group 1 vs group 3	-3.82	0.488
	Group 1 vs group 2	-0.78	0.913
	Group 2 vs group 3	-2.44	0.721
HF power	Group 1 vs group 3	20.4	0.001***
	Group 1 vs group 2	8.51	0.752
	Group 2 vs group 3	4.54	0.772
LF power	Group 1 vs group 3	-2.15	0.701
	Group 1 vs group 2	-1.1	0.881
	Group 2 vs group 3	1.11	0.879
LF/HF ratio	Group 1 vs group 3	-0.843	0.002**
	Group 1 vs group 2	0.29	0.889
	Group 2 vs group 3	-0.131	0.935

## Discussion

The present study investigated the effects of two interventions - cold spinal spray and cold spinal bath, compared with supine rest - on cognitive performance and cardiac autonomic modulation. Cognitive outcomes were assessed using the Stroop task and Digit Letter Substitution Test (DLST); HRV indices provided physiological insights into autonomic regulation. The results reveal significant time effects, group-specific trends, and meaningful improvements, particularly in correct responses, RMSSD, and LF/HF ratio, supporting the efficacy of cold hydrotherapy modalities in enhancing both cognitive and physiological functioning.

The increase in correct responses from pre- to post-intervention suggests better attention and reaction time in both the cold spinal spray groups. Also, both the intervention groups improved the accuracy and reduced the error rate, indicating a general enhancement in task performance. These results are similar to what has been observed in a study, where a small sample of healthy adult participants (approximately 8-12 individuals) were exposed to controlled cold conditions for one hour, which has proven to be an effective intervention in improving the cognitive functions in healthy young women [26]. However, neither intervention could bring down the Stroop effect (reaction time difference between congruent and incongruent conditions), suggesting that deeper cognitive interference mechanisms may be more resistant to short-term intervention.

The number of correct responses was higher in the cold spinal spray group, although no such improvement can be seen in accuracy and error rate in that group; however, cold spinal bath improved the accuracy and reduced the error rate when compared to both cold spinal spray and the control group, suggesting improved attentional control. Also, compared to other groups, group 1 showed a reduction in Stroop interference, pointing to enhanced cognitive control and processing speed. In contrast, group 3 showed a post-intervention increase in both errors and Stroop effect values, indicating potential inefficiency in cognitive conflict resolution.

Overall, these results indicate that cognitive performance improved over time, but the extent of improvement varied by intervention. The cold spinal spray appeared to be most effective in enhancing response speed and conflict management, while the cold spinal bath showed better results in accuracy and error suppression, suggesting distinct mechanisms of action. The absence of significant change in the Stroop effect across groups further suggests that cognitive interference processing may require more sustained or targeted interventions. The possible mechanism behind it could be the release of dopamine that helps transmit messages to the reward systems in our brain and plays a critical role in planning, task completion, and learning, as from previous research we know plasma norepinephrine increased by 530%, and dopamine increases by 250% during immersion in 14 °C water, demonstrating a substantial neurochemical response to acute cold exposure, and as it is already evidenced that dopamine activity in the prefrontal cortex modulates planning, decision-making, and focused attention, making it essential for task execution and cognitive flexibility [27-28].

Heart rate variability analysis revealed that both cold interventions significantly improved autonomic regulation. Group 1 exhibited changes both in the time and frequency domains of HRV, indicating a substantial shift towards parasympathetic dominance. This is consistent with literature showing that localized cold exposure can stimulate cutaneous and spinal afferents, enhancing vagal activity [29-30].

Group 2 also improved in time domains of HRV, though to a lesser extent. The broader surface exposure of the immersion technique may provide more general thermal stimulation, but possibly with less intense afferent activation. In contrast, group 3 showed either stagnation or deterioration in HRV, showing significant changes suggesting sympathetic dominance, reinforcing the need for active physiological stimulation to restore autonomic balance. A similar study done on healthy individuals to explore the effect of cold spinal spray and cold spinal bath showed comparable results, which further substantiate the findings of the current research [13].

The randomized controlled study examined the immediate effects of cold spinal spray and cold spinal bath on cognitive and autonomic function in young adults. Both hydrotherapy methods significantly enhanced cognitive performance and heart rate variability compared to rest, with cold spinal bath improving correct responses and cold spinal spray improving accuracy and parasympathetic activity, supporting hydrotherapy’s potential for cognitive enhancement and stress recovery applications. A major strength of the study is the randomized controlled design with blinded outcome assessment, enhancing the validity and reliability of the results. The study uses objective, validated cognitive and HRV measures, and rigorous methodological controls (e.g., standardized intervention duration, water temperature, resting controls). Although there are several strengths of this study, there are a few limitations, which include the inability to assess the long-term effect of the treatments due to the immediate nature of the study, not being multi-centred, not being blinded because of the nature of the treatments, and unequal distribution of gender in the sample. Future research should use longer interventions, diverse samples, neuroimaging, and biochemical markers, and target clinical populations.

## Conclusions

Cold spinal hydrotherapy, especially spinal spray, produced marked short-term cognitive and autonomic improvements in healthy adults, with enhanced cognitive performance and HRV. These safe, simple interventions deserve consideration in naturopathic protocols for cognitive enhancement and stress recovery, offering promising adjunctive benefits in preventive and therapeutic contexts.

## References

[1] Dhakal A, Bobrin BD (2025). Cognitive deficits. https://www.ncbi.nlm.nih.gov/books/NBK559052/.

[2] Mesulam MM (1998). From sensation to cognition. Brain.

[3] Harvey PD (2019). Domains of cognition and their assessment. Dialogues Clin Neurosci.

[4] Jia J, Zhao T, Liu Z (2023). Association between healthy lifestyle and memory decline in older adults: 10 year, population based, prospective cohort study. BMJ.

[5] Dinesh S (2014). Immediate hypoglycaemic effect of two selective hydrotherapeutic procedures in non-insulin-dependent patients with diabetes mellitus. J Res Educ Indian Med.

[6] Mooventhan A, Nivethitha L (2014). Scientific evidence-based effects of hydrotherapy on various systems of the body. N Am J Med Sci.

[7] Mäkinen TM, Palinkas LA, Reeves DL, Pääkkönen T, Rintamäki H, Leppäluoto J, Hassi J (2006). Effect of repeated exposures to cold on cognitive performance in humans. Physiol Behav.

[8] Knill-Jones J, Shadwell G, Hurst HT, Mawhinney C, Sinclair JK, Allan R (2025). Influence of acute and chronic therapeutic cooling on cognitive performance and well-being. Physiol Behav.

[9] Adkins M, Cox R, Axelsson J (2023). Impact of cold-water hand immersion on cognitive performance and sleepiness during sleep inertia. Sleep.

[10] Jones DM, Bailey SP, De Pauw K, Folger S, Roelands B, Buono MJ, Meeusen R (2019). Evaluation of cognitive performance and neurophysiological function during repeated immersion in cold water. Brain Res.

[11] González-Ibarra FP, Varon J, López-Meza EG (2011). Therapeutic hypothermia: critical review of the molecular mechanisms of action. Front Neurol.

[12] Yang HJ, Shi X, Ju F (2018). Cold shock induced protein RBM3 but not mild hypothermia protects human SH-SY5Y neuroblastoma cells from MPP+-induced neurotoxicity. Front Neurosci.

[13] Vidyasagar Vidyasagar, Geetha KV (2019). Comparative study on effect of cold spinal spray and cold spinal bath on autonomic variables in healthy individuals. J Emerg Technol Innov Res.

[14] Shetty B, Shetty GB, Shetty P (2016). Immediate effect of cold spinal bath on autonomic and respiratory variables in hypertensives. World J Pharm Med Res.

[15] Gowda M, KJ S, Shetty P (2020). Physiological effects of short and prolonged cold spinal Bath on heart rate variability and respiratory rate in healthy volunteers. J Res Tradit Med.

[16] Deshpande S, Nagendra HR, Raghuram N (2008). A randomized control trial of the effect of yoga on verbal aggressiveness in normal healthy volunteers. Int J Yoga.

[17] Jainraj R, Nair PMK, Khawale P (2016). Immediate effect of cool spinal bath on blood pressure of healthy volunteers - results of a single arm study. Adv Integr Med.

[18] Yildirir A, Kabakci G, Akgul E, Tokgozoglu L, Oto A (2002). Effects of menstrual cycle on cardiac autonomic innervation as assessed by heart rate variability. Ann Noninvasive Electrocardiol.

[19] Boutron I, Altman DG, Moher D, Schulz KF, Ravaud P (2017). CONSORT statement for randomized trials of nonpharmacologic treatments: a 2017 update and a CONSORT extension for nonpharmacologic trial Abstracts. Ann Intern Med.

[20] Kellogg JH (1918). Rational Hydrotherapy: A Manual of the Physiological and Therapeutic Effects of Hydriatic Procedures, and the Technique of Their Application in the Treatment of Disease.

[21] Avanthika G (2017). Immediate effect of cold and neutral spinal spray on autonomic functions in healthy volunteers - a comparative study. IOSR J Dent Med Sci.

[22] (2017). Inquisit version 7. https://www.millisecond.com/download.

[23] Scarpina F, Tagini S (2017). The Stroop Color and Word Test. Front Psychol.

[24] Falla M, Micarelli A, Hüfner K, Strapazzon G (2021). The effect of cold exposure on cognitive performance in healthy adults: a systematic review. Int J Environ Res Public Health.

[25] Shaffer F, Ginsberg JP (2017). An overview of heart rate variability metrics and norms. Front Public Health.

[26] Enander A (1987). Effects of moderate cold on performance of psychomotor and cognitive tasks. Ergonomics.

[27] Srámek P, Simecková M, Janský L, Savlíková J, Vybíral S (2000). Human physiological responses to immersion into water of different temperatures. Eur J Appl Physiol.

[28] Bromberg-Martin ES, Matsumoto M, Hikosaka O (2010). Dopamine in motivational control: rewarding, aversive, and alerting. Neuron.

[29] Shetty P, Sujatha KJ, Mooventhan A, Shashikiran HC, Yalla D, Thayill J, Arun PG (2024). Effect of a yoga and naturopathy-based lifestyle intervention with nine-month follow-up on heart rate variability in patients with hypertension: a randomized controlled trial. J Bodyw Mov Ther.

[30] Chang RB (2019). Body thermal responses and the vagus nerve. Neurosci Lett.

